# Crystal structure of a Pd_4_ carbonyl tri­phenyl­phosphane cluster [Pd_4_(CO)_5_(PPh_3_)_4_]·2C_4_H_8_O, comparing solvates

**DOI:** 10.1107/S205698901502441X

**Published:** 2016-01-06

**Authors:** Koen Robeyns, Christopher Willocq, Bernard Tinant, Michel Devillers, Sophie Hermans

**Affiliations:** a Institute of Condensed Matter and Nanosciences (IMCN), Université Catholique de Louvain, 1 Place Louis Pasteur, B 1348 Louvain-la-Neuve, Belgium

**Keywords:** structure transformation, homonuclear cluster, palladium, gold, crystal structure

## Abstract

Attempts to synthesize Au–Pd heterometallic compounds from homonuclear palladium or gold complexes, [Pd(PtBu_2_)_2_] and [Au(PPh_3_)Cl] in a tetra­hydro­furan (THF) solution under a CO atmosphere resulted in a homonuclear Pd cluster, namely penta­kis­(μ-carbonyl-κ^2^ *C*:*C*)tetra­kis­(tri­phenyl­phosphane-κ*P*)tetrapalladium(5 *Pd*—*Pd*) tetra­hydro­furan disolvate, [Pd_4_(CO)_5_(C_18_H_15_P)_4_]·2C_4_H_8_O. The complex mol­ecule lies on a twofold rotation axis. The crystal structure is described in relation to the CH_2_Cl_2_ solvate previously determined by our group [Willocq *et al.* (2011 ▸). *Inorg. Chim. Acta*, **373**, 233–242], and in particular to the desolvated structure [Feltham *et al.* (1985 ▸). *Inorg. Chem.* **24**, 1503–1510]. It is assumed that the title compound transforms into the latter structure, upon gradual loss of solvent mol­ecules. In the title compound, the symmetry-unique THF solvent mol­ecule is linked to the complex mol­ecule by a weak C—H⋯O hydrogen bond. Contributions of disordered solvent molecules to the diffraction intensities, most likely associated with methanol, were removed with the SQUEEZE [Spek (2015). *Acta Cryst.* C**71**, 9–18] algorithm.

## Abstract 2

The reported homonuclear Pd_4_ cluster is presented in relation to structural analogues. Gradual evaporation of the trapped solvent mol­ecules results in a unilateral contraction of the unit cell, transforming it into the solvent-free structure.

## Chemical context   

Heterometallic compounds are ideal precursors for mixed oxides or mixed-metal nanoparticles, especially when the two considered metals are difficult to alloy. In the case of the Pd–Au combination, a tremendous amount of work has been carried out recently in heterogeneous catalysis to prepare supported bimetallic catalysts with a fine control over composition and size of the supported heterometal nanoparticles (Paalanen *et al.*, 2013 ▸). These materials find, for example, application in the direct synthesis of hydrogen peroxide from hydrogen and oxygen (Edwards *et al.*, 2015 ▸), or liquid-phase oxidation of alcohols and aldehydes (Villa *et al.*, 2015 ▸; Hermans & Devillers, 2005 ▸; Hermans *et al.*, 2010 ▸, 2011 ▸). However, synthesizing mol­ecular compounds presenting a hetero metal–metal bond is challenging. Several strategies have been described, such as reactions of metal salts in the presence of a reducing agent or reactions under irradiation (favoring formation of metal–metal bonds). In the present work, we explore the reactivity of Au and Pd compounds in a CO atmosphere, with the hope of providing the reducing agent and additional ligands through dissolved carbon monoxide. The direct synthesis of Au–Pd heterometallic complexes has already been achieved using similar strategies, for example starting from [Pd(PPh_3_)_2_Cl_2_] and [Au(PPh_3_)NO_3_] in the presence of NaBH_4_ (Ito *et al.*, 1991 ▸; Quintilio *et al.*, 1994 ▸). One major drawback of this type of strategy is that the product formed is unpredictable, with easy cluster formation by aggregation and homometal bond formation. We have devised in parallel a more predictable synthesis method for Au–Pd compounds by adding a cationic Au fragment to a reduced Pd species (Willocq *et al.*, 2011 ▸). Here we describe a homometallic Pd_4_ cluster formed by reductive carbonyl­ation and coalescence of a Pd complex in presence of an Au phosphine compound. The reported cluster is closely related to known Pd_4_ cluster structures (Willocq *et al.*, 2011 ▸; Mednikov *et al.*, 1987 ▸; Feltham *et al.*, 1985 ▸).
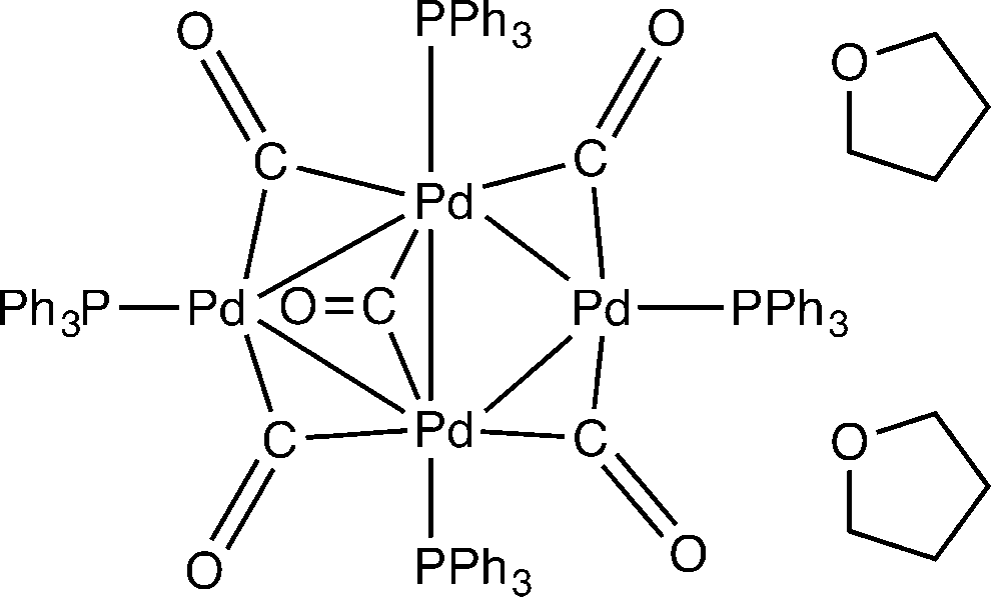


## Structural commentary   

**Figure 1:**
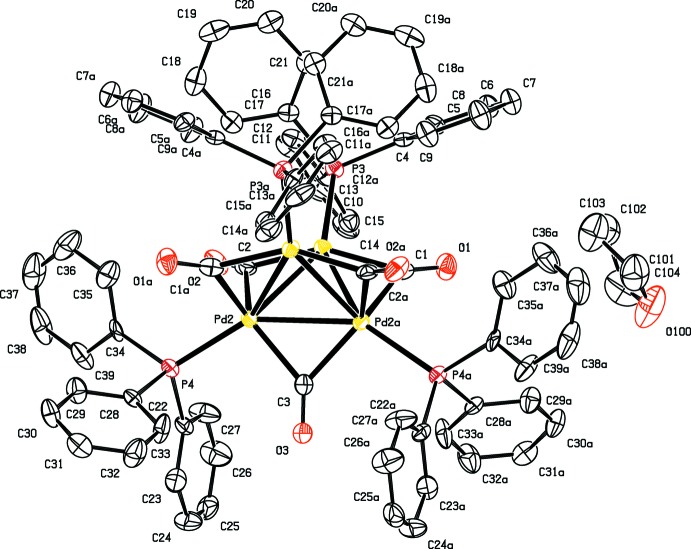
Molecular structure of the title compound, showing displacement ellipsoids drawn at the 50% probability level. [Symmetry code: (*a*) −*x*, *y*, −*z* + 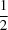.] Only the symmetry-unique THF solvent mol­ecule is shown.

The structure of the Pd cluster (Fig. 1 ▸) shows inter­nal symmetry and is located on a twofold rotation axis, passing through the central carbonyl, giving four complex mol­ecules in the unit cell (*Z*′ = 0.5). Crystallized from a THF/MeOH mixture, the reported structure is a THF solvate, revealing eight tetra­hydro­furan mol­ecules in the unit cell. Around the inversion centres, 60 Å cavities are located which were treated by the *PLATON* (Spek, 2009 ▸) SQUEEZE (Spek, 2015 ▸) algorithm, accounting to 15 electrons. A single peak, on the special position, was visible in this cavity, which is believed to be the oxygen atom of a partially occupied and disordered MeOH mol­ecule. Partial evaporation of the solvent mol­ecules probably explains the limited resolution of the collected data. Reflection data up to 0.94 Å were used during refinement, this being the best diffracting crystal amongst several tested.

The central unit of the complex consists of four Pd atoms at the corners of a distorted tetra­hedron. Of the six edges, five are occupied by bridging carbonyl ligands, the remaining one has a non-bonding Pd⋯Pd distance of 3.170 (1) Å. The bonding Pd—Pd distances are in the range 2.7381 (8)–2.8006 (12) Å (Table 1 ▸). The same compound had been crystallized earlier by our group (Willocq *et al.*, 2011 ▸) as a CH_2_Cl_2_ solvate in the triclinic space group *P* 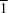. The mol­ecular geometry of both structures is quite different, the most pronounced difference being the lack of inter­nal symmetry in the *P* 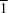 structure, which can be extended to the symmetry of the Pd core. The Pd—Pd distances opposite the non-bonding Pd—Pd are very similar, 2.801 (1) and 2.805 (1) Å (*P* 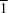). Although the average of the four remaining Pd—Pd bond lengths in the two structures is quite similar (2.741 Å for the current structure and 2.746 Å for the triclinic structure), the bond-length distribution is quite different, showing equal bond lengths for the current structure and two shorter [2.678 (1) and 2.720 (1) Å] and two longer ones [2.797 (1) and 2.790 (1) Å] for the triclinic structure.

No classical hydrogen-bond inter­actions are observed, but a weak C—H ⋯O inter­action (Table 2 ▸) can be considered to the oxygen atom of the THF mol­ecule.

## Database survey   

A survey of the Cambridge Structural Database (Groom & Allen, 2014 ▸) revealed two more occurrences of the title compound, both crystallized in the *C*2/c space group. In the paper by Mednikov *et al.* (1987 ▸) the homonuclear Pd cluster is reported as a co-former, together with a trinuclear Pd cluster [Pd_3_(CO)_3_(PPh_3_)_4_], here as well the Pd cluster is found onto a twofold rotation axis and superposition of both mol­ecular entities reveals similar features, right up to similar orientations of the tri­phenyl­phosphines.

The second occurrence is however much more inter­esting as the structure of Feltham *et al.* (1985 ▸) shows remarkable similarities with the reported structure, not only with respect to the mol­ecular conformation – the r.m.s. deviation between the two structures is 0.757 Å for all atoms, and 0.356 Å when omitting the phenyl rings – but also with respect to the overall crystal packing. Closer inspection of the unit-cell parameters, listed below, reveals that for both structures only the *a* axis differs significantly by more than 2 Å (2.297 Å):

*a* = 27.254 (9), *b* = 16.016 (6), *c* = 17.938 (7) Å, β = 105.92 (2)°, *V* = 7530.0 Å^3^, 120 K (title compound);

*a* = 24.957 (5), *b* = 16.138 (3), *c* = 17.758 (3) Å, β = 103.47 (2)°, *V* = 6955.4 Å^3^, RT, (Feltham *et al.*, 1985 ▸).

While the Feltham *et al.* (1985 ▸) structure contains a total of 400 Å^3^ of voids distributed over six sites (pore sizes from 5–32 Å^3^), none of these is big enough to host even small solvent mol­ecules, characterizing this structure as solvent-free. Gradual loss of solvent mol­ecules is believed to provoke a transformation from the solvent-rich title compound to the desolvated structure reported by Feltham *et al.* (1985 ▸). The reported problems during crystal harvesting of the latter structure (see section 4) tends to support this hypothesis. The transformation itself appears to occur in a sequential process where two types of solvent cavities gradually lose their solvent mol­ecules, leading to a contraction of the *a* axis. The first affected cavities are the 61 Å^3^ voids treated by SQUEEZE (Spek, 2015 ▸) in the current structure, followed by the cavity hosting the loosely trapped THF mol­ecule (289 Å^3^). After correcting for the inter­stitial voids observed in the contracted structure, the volume loss during the transformation is in complete agreement with the solvent loss in both cavities.

**Figure 2:**
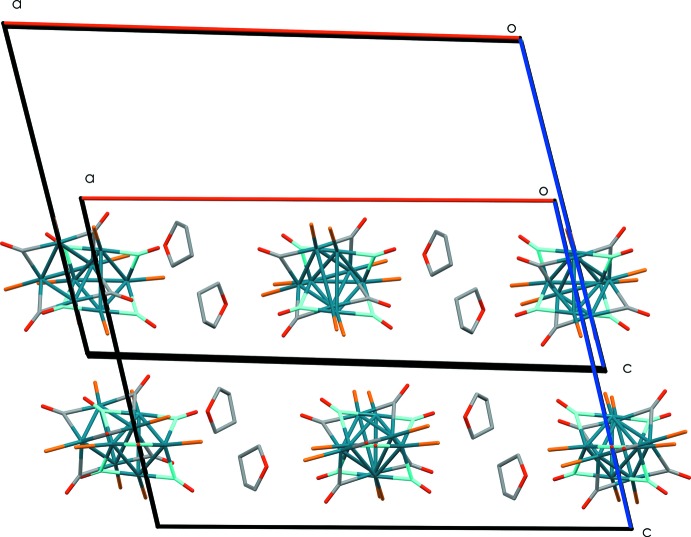
Packing overlay of the title compound measured at 120 K and the solvent-free structure of Feltham *et al.* (1985 ▸) measured at room temperature, obtained by pairwise fitting of all Pd atoms of the four mol­ecules in the unit cell. The projection along the *b* axis reveals that, upon evaporation of the solvent mol­ecules, the unit cell contracts, while keeping the global packing arrangement. Phenyl rings have been omitted for clarity.

Fig. 2 ▸ shows the packing overlay by pairwise fitting of the Pd atoms of the reported structure and the structure of Feltham *et al.* (1985 ▸) (all Pd atoms within one unit cell were considered); evaporation of the THF mol­ecules and small rearrangements of the homonuclear cluster allows the transformation of the solvated structure into the solvent-free analogue to be completed. This transformation only involves one dimension and a projection along the *a* axis of the superimposed unit cells reveals practically fully overlapped mol­ecules, even when considering the orientation of the phenyl rings.

## Synthesis and crystallization   

The synthesis of the title compound was an attempt to obtain mixed Au–Pd complexes in a one-step reaction. Through a THF solution of [Pd(P^t^Bu_3_)_2_] and [Au(PPh_3_)Cl] carbon monoxide gas was passed and the solid material left after evaporation of the solvent was characterized by NMR and IR spectroscopy. One intense IR band at 1870 cm^−1^ indicated the formation of a complex with CO ligands. ^31^P NMR showed two signals at 28.1 and 97.2 p.p.m. with a 4:1 ratio, which indicate the presence of two types of phosphines, while the ^1^H NMR indicated the presence of both tri­phenyl­phosphine and tri-*tert*-butyl­phosphine. Dissolution of the solid in a THF/MeOH mixture yielded red crystals which were suitable for X-ray diffraction. Rather than a mixed Au–Pd species, the crystals contained a homonuclear Pd complex.

Previously the synthesis of the title compound was reported as the reduction of an oxygen-free CH_2_Cl_2_ solution of [Pd(NO_2_)_2_(PPh_3_)_3_] under CO. Crystals were formed upon cooling after addition of CO-saturated hexane and were reported to decompose rapidly and could finally be measured at room temperature in a CO-filled sealed capillary (Feltham *et al.*, 1985 ▸). The homonuclear Pd_4_ cluster can also be synthesized by the reaction of [Pd_2_(dba)_3_] (dba is dibenzyl­idene­acetone) and three equivalents of PPh_3_ under CO (Willocq *et al.*, 2011 ▸).

## Refinement   

Crystal data and structure refinement details are summarized in Table 3 ▸. Data were collected on a MAR345 image plate, using Mo Kα radiation generated on a Rigaku UltraX 18S generator (Zr filter). Diffaction data were not corrected for absorption, but the data collection mode with high redundancy, partially takes the absorption phenomena into account. (111 images, ΔΦ = 2°, 21617 reflections measured for 4740 independent reflections). H atoms were placed at calculated positions with isotropic temperature factors fixed at 1.2*U*_eq_ of the parent atom.

## Supplementary Material

Crystal structure: contains datablock(s) I, New_Global_Publ_Block. DOI: 10.1107/S205698901502441X/lh5801sup1.cif

Structure factors: contains datablock(s) I. DOI: 10.1107/S205698901502441X/lh5801Isup2.hkl

Supporting information file. DOI: 10.1107/S205698901502441X/lh5801Isup3.cdx

CCDC reference: 1443364

Additional supporting information:  crystallographic information; 3D view; checkCIF report

## Figures and Tables

**Table 1:** Selected bond lengths (Å)

Pd1—Pd2	2.7381 (8)	Pd2—P4	2.3208 (15)
Pd1—Pd2^i^	2.7446 (9)	Pd2—Pd1^i^	2.7446 (9)
Pd1—Pd1^i^	3.1704 (14)	Pd2—Pd2^i^	2.8006 (12)

Symmetry code: (i) 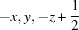.

**Table 2:** Hydrogen-bond geometry (Å, °)

*D*—H⋯*A*	*D*—H	H⋯*A*	*D*⋯*A*	*D*—H⋯*A*
C19—H19⋯O100^ii^	0.95	2.43	3.282 (8)	149

Symmetry code: (ii) 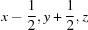.

**Table 3:** Experimental details

Crystal data	
Chemical formula	[Pd_4_(CO)_5_(C_18_H_15_P)_4_]·2C_4_H_8_O
*M*_r_	1758.93
Crystal system, space group	Monoclinic, *C*2/*c*
Temperature (K)	120
*a*, *b*, *c* (Å)	27.254 (9), 16.016 (6), 17.938 (7)
β (°)	105.92 (2)
*V* (Å^3^)	7530 (5)
*Z*	4
Radiation type	Mo *K*α
μ (mm^−1^)	1.08
Crystal size (mm)	0.18 × 0.12 × 0.05

Data collection	
Diffractometer	MAR345 image plate
No. of measured, independent and observed [*I* > 2σ(*I*)] reflections	9236, 4739, 3938
*R*_int_	0.037
θ_max_ (°)	22.2
(sin θ/λ)_max_ (Å^−1^)	0.532

Refinement	
*R*[*F* ^2^ > 2σ(*F* ^2^)], *wR*(*F* ^2^), *S*	0.035, 0.086, 1.04
No. of reflections	4739
No. of parameters	453
H-atom treatment	H-atom parameters constrained
Δρ_max_, Δρ_min_ (e Å^−3^)	0.54, −0.53

Computer programs: *mar345* (Klein, 1998 ▸), *marHKL* (Klein & Bartels, 2000 ▸), *SHELXS97* (Sheldrick, 2008 ▸), *SHELXL2014* (Sheldrick, 2015 ▸), *Mercury* (Macrae *et al.*, 2008 ▸) and *PLATON* (Spek, 2009 ▸).

## supplementary crystallographic information

### Crystal data

**Table:**

[Pd_4_(CO)_5_(C_18_H_15_P)_4_]·2C_4_H_8_O	*F*(000) = 3544
*M**_r_* = 1758.93	*D*_x_ = 1.552 Mg m^−^^3^
Monoclinic, *C*2/*c*	Mo *K*α radiation, λ = 0.71069 Å
*a* = 27.254 (9) Å	Cell parameters from 4740 reflections
*b* = 16.016 (6) Å	θ = 2.6–22.2°
*c* = 17.938 (7) Å	µ = 1.08 mm^−^^1^
β = 105.92 (2)°	*T* = 120 K
*V* = 7530 (5) Å^3^	Platelets, red
*Z* = 4	0.18 × 0.12 × 0.05 mm

### Data collection

**Table:**

MAR345 image plate diffractometer	3938 reflections with *I* > 2σ(*I*)
Radiation source: Rigaku UltraX 18 rotating anode	*R*_int_ = 0.037
Zr filter monochromator	θ_max_ = 22.2°, θ_min_ = 2.5°
111 images, ΔΦ 2° scans	*h* = −28→28
9236 measured reflections	*k* = −17→17
4739 independent reflections	*l* = −19→19

### Refinement

**Table:**

Refinement on *F*^2^	0 restraints
Least-squares matrix: full	Hydrogen site location: inferred from neighbouring sites
*R*[*F*^2^ > 2σ(*F*^2^)] = 0.035	H-atom parameters constrained
*wR*(*F*^2^) = 0.086	*w* = 1/[σ^2^(*F*_o_^2^) + (0.0201*P*)^2^ + 36.9627*P*] where *P* = (*F*_o_^2^ + 2*F*_c_^2^)/3
*S* = 1.03	(Δ/σ)_max_ = 0.001
4739 reflections	Δρ_max_ = 0.54 e Å^−^^3^
453 parameters	Δρ_min_ = −0.53 e Å^−^^3^

### Special details

**Table:**

Geometry. All e.s.d.'s (except the e.s.d. in the dihedral angle between two l.s. planes) are estimated using the full covariance matrix. The cell e.s.d.'s are taken into account individually in the estimation of e.s.d.'s in distances, angles and torsion angles; correlations between e.s.d.'s in cell parameters are only used when they are defined by crystal symmetry. An approximate (isotropic) treatment of cell e.s.d.'s is used for estimating e.s.d.'s involving l.s. planes.

### Fractional atomic coordinates and isotropic or equivalent isotropic displacement parameters (Å^2^)

**Table:**

	*x*	*y*	*z*	*U*_iso_*/*U*_eq_	
Pd1	0.00088 (2)	0.70651 (2)	0.16201 (2)	0.01857 (13)	
Pd2	−0.05342 (2)	0.59762 (2)	0.22629 (2)	0.01713 (13)	
C1	0.0757 (2)	0.6757 (3)	0.1897 (3)	0.0248 (13)	
O1	0.11225 (15)	0.6829 (2)	0.1692 (2)	0.0345 (9)	
C2	−0.0735 (2)	0.6741 (3)	0.1236 (3)	0.0239 (12)	
O2	−0.10823 (14)	0.6805 (2)	0.0691 (2)	0.0333 (9)	
C3	0.0000	0.5003 (5)	0.2500	0.0231 (17)	
O3	0.0000	0.4268 (3)	0.2500	0.0320 (13)	
P3	−0.00131 (5)	0.82666 (8)	0.09097 (7)	0.0185 (3)	
C4	0.06148 (19)	0.8732 (3)	0.1019 (3)	0.0180 (11)	
C5	0.0797 (2)	0.8963 (3)	0.0400 (3)	0.0235 (12)	
H5	0.0579	0.8915	−0.0113	0.028*	
C6	0.1284 (2)	0.9258 (3)	0.0510 (3)	0.0275 (13)	
H6	0.1405	0.9401	0.0078	0.033*	
C7	0.1599 (2)	0.9346 (4)	0.1258 (3)	0.0328 (14)	
H7	0.1937	0.9548	0.1340	0.039*	
C8	0.1422 (2)	0.9140 (4)	0.1878 (3)	0.0365 (15)	
H8	0.1636	0.9211	0.2390	0.044*	
C9	0.0937 (2)	0.8833 (3)	0.1764 (3)	0.0281 (13)	
H9	0.0819	0.8686	0.2199	0.034*	
C10	−0.02718 (19)	0.8146 (3)	−0.0143 (3)	0.0208 (12)	
C11	−0.0615 (2)	0.8691 (3)	−0.0601 (3)	0.0262 (13)	
H11	−0.0741	0.9150	−0.0372	0.031*	
C12	−0.0777 (2)	0.8572 (4)	−0.1398 (3)	0.0346 (15)	
H12	−0.1024	0.8936	−0.1714	0.041*	
C13	−0.0577 (2)	0.7919 (4)	−0.1732 (3)	0.0373 (15)	
H13	−0.0678	0.7850	−0.2279	0.045*	
C14	−0.0234 (2)	0.7371 (4)	−0.1274 (3)	0.0373 (15)	
H14	−0.0100	0.6924	−0.1506	0.045*	
C15	−0.0085 (2)	0.7470 (3)	−0.0478 (3)	0.0280 (13)	
H15	0.0143	0.7082	−0.0161	0.034*	
C16	−0.03973 (19)	0.9103 (3)	0.1160 (3)	0.0199 (12)	
C17	−0.0907 (2)	0.8940 (3)	0.1112 (3)	0.0253 (13)	
H17	−0.1050	0.8410	0.0939	0.030*	
C18	−0.1208 (2)	0.9551 (4)	0.1318 (3)	0.0317 (14)	
H18	−0.1556	0.9436	0.1275	0.038*	
C19	−0.1010 (2)	1.0321 (3)	0.1581 (3)	0.0327 (15)	
H19	−0.1216	1.0734	0.1728	0.039*	
C20	−0.0505 (2)	1.0477 (4)	0.1628 (3)	0.0309 (14)	
H20	−0.0364	1.1008	0.1802	0.037*	
C21	−0.0199 (2)	0.9878 (3)	0.1425 (3)	0.0258 (13)	
H21	0.0149	0.9998	0.1468	0.031*	
P4	−0.12826 (5)	0.52022 (8)	0.19235 (7)	0.0191 (3)	
C22	−0.12432 (19)	0.4330 (3)	0.1291 (3)	0.0233 (12)	
C23	−0.1279 (2)	0.3498 (3)	0.1471 (3)	0.0314 (14)	
H23	−0.1329	0.3344	0.1956	0.038*	
C24	−0.1241 (2)	0.2883 (4)	0.0939 (4)	0.0433 (16)	
H24	−0.1259	0.2312	0.1072	0.052*	
C25	−0.1178 (2)	0.3084 (4)	0.0231 (4)	0.0373 (15)	
H25	−0.1160	0.2660	−0.0131	0.045*	
C26	−0.1141 (3)	0.3903 (4)	0.0058 (4)	0.0475 (17)	
H26	−0.1096	0.4052	−0.0431	0.057*	
C27	−0.1170 (2)	0.4521 (4)	0.0578 (3)	0.0419 (16)	
H27	−0.1139	0.5088	0.0444	0.050*	
C28	−0.14892 (18)	0.4741 (3)	0.2725 (3)	0.0195 (12)	
C29	−0.1977 (2)	0.4841 (4)	0.2804 (3)	0.0312 (14)	
H29	−0.2223	0.5150	0.2426	0.037*	
C30	−0.2109 (2)	0.4494 (4)	0.3431 (3)	0.0359 (15)	
H30	−0.2441	0.4583	0.3491	0.043*	
C31	−0.1761 (2)	0.4022 (3)	0.3967 (3)	0.0323 (14)	
H31	−0.1856	0.3765	0.4384	0.039*	
C32	−0.1277 (2)	0.3926 (4)	0.3896 (3)	0.0411 (16)	
H32	−0.1034	0.3604	0.4266	0.049*	
C33	−0.1139 (2)	0.4299 (4)	0.3281 (3)	0.0349 (15)	
H33	−0.0798	0.4247	0.3246	0.042*	
C34	−0.18558 (19)	0.5748 (3)	0.1361 (3)	0.0232 (12)	
C35	−0.1923 (2)	0.6592 (4)	0.1488 (3)	0.0359 (15)	
H35	−0.1663	0.6881	0.1861	0.043*	
C36	−0.2352 (2)	0.7020 (4)	0.1092 (4)	0.0501 (18)	
H36	−0.2390	0.7594	0.1197	0.060*	
C37	−0.2727 (3)	0.6609 (5)	0.0542 (4)	0.057 (2)	
H37	−0.3024	0.6902	0.0263	0.069*	
C38	−0.2673 (2)	0.5785 (5)	0.0398 (4)	0.053 (2)	
H38	−0.2933	0.5506	0.0016	0.064*	
C39	−0.2241 (2)	0.5345 (4)	0.0806 (3)	0.0374 (15)	
H39	−0.2209	0.4769	0.0704	0.045*	
O100	0.2944 (2)	0.6292 (4)	0.1655 (3)	0.0822 (18)	
C101	0.2603 (3)	0.6561 (5)	0.0954 (5)	0.079 (3)	
H10A	0.2303	0.6185	0.0805	0.095*	
H10B	0.2773	0.6562	0.0533	0.095*	
C102	0.2439 (3)	0.7435 (5)	0.1096 (4)	0.063 (2)	
H10C	0.2675	0.7859	0.0989	0.076*	
H10D	0.2088	0.7555	0.0777	0.076*	
C103	0.2467 (3)	0.7403 (5)	0.1944 (4)	0.069 (2)	
H10E	0.2541	0.7962	0.2185	0.082*	
H10F	0.2143	0.7194	0.2024	0.082*	
C104	0.2894 (3)	0.6812 (5)	0.2274 (4)	0.063 (2)	
H10G	0.3215	0.7123	0.2497	0.076*	
H10H	0.2818	0.6471	0.2689	0.076*	

### Atomic displacement parameters (Å^2^)

**Table:**

	*U*^11^	*U*^22^	*U*^33^	*U*^12^	*U*^13^	*U*^23^
Pd1	0.0184 (2)	0.0189 (2)	0.0177 (2)	−0.00139 (17)	0.00377 (16)	0.00374 (17)
Pd2	0.0165 (2)	0.0166 (2)	0.0186 (2)	−0.00199 (16)	0.00526 (16)	0.00040 (17)
C1	0.035 (4)	0.020 (3)	0.017 (3)	0.002 (3)	0.002 (3)	0.000 (2)
O1	0.030 (2)	0.044 (3)	0.035 (2)	0.0032 (19)	0.0176 (19)	0.0124 (19)
C2	0.032 (3)	0.014 (3)	0.029 (3)	−0.001 (2)	0.014 (3)	0.004 (2)
O2	0.028 (2)	0.038 (2)	0.026 (2)	−0.0103 (18)	−0.0057 (19)	0.0121 (18)
C3	0.021 (4)	0.026 (5)	0.024 (4)	0.000	0.008 (3)	0.000
O3	0.021 (3)	0.025 (3)	0.048 (4)	0.000	0.006 (2)	0.000
P3	0.0200 (7)	0.0193 (7)	0.0157 (7)	−0.0018 (6)	0.0038 (5)	0.0031 (6)
C4	0.023 (3)	0.012 (3)	0.019 (3)	0.002 (2)	0.005 (2)	0.002 (2)
C5	0.026 (3)	0.022 (3)	0.022 (3)	−0.004 (2)	0.006 (2)	0.004 (2)
C6	0.029 (3)	0.032 (3)	0.023 (3)	−0.002 (3)	0.010 (2)	0.006 (3)
C7	0.027 (3)	0.040 (4)	0.033 (3)	−0.006 (3)	0.010 (3)	0.001 (3)
C8	0.029 (4)	0.052 (4)	0.025 (3)	−0.012 (3)	0.003 (3)	−0.001 (3)
C9	0.029 (3)	0.040 (3)	0.016 (3)	−0.009 (3)	0.006 (2)	0.002 (3)
C10	0.021 (3)	0.025 (3)	0.017 (3)	−0.007 (2)	0.006 (2)	0.004 (2)
C11	0.031 (3)	0.022 (3)	0.023 (3)	−0.005 (2)	0.003 (2)	0.002 (2)
C12	0.037 (4)	0.033 (3)	0.027 (3)	−0.008 (3)	−0.001 (3)	0.007 (3)
C13	0.054 (4)	0.034 (4)	0.025 (3)	−0.023 (3)	0.014 (3)	−0.009 (3)
C14	0.045 (4)	0.033 (4)	0.036 (4)	−0.016 (3)	0.015 (3)	−0.014 (3)
C15	0.027 (3)	0.032 (3)	0.027 (3)	−0.003 (3)	0.010 (2)	−0.004 (3)
C16	0.025 (3)	0.020 (3)	0.013 (3)	0.001 (2)	0.002 (2)	0.003 (2)
C17	0.028 (3)	0.029 (3)	0.017 (3)	−0.003 (3)	0.003 (2)	0.000 (2)
C18	0.027 (3)	0.051 (4)	0.018 (3)	0.009 (3)	0.006 (2)	0.008 (3)
C19	0.055 (4)	0.029 (4)	0.014 (3)	0.014 (3)	0.009 (3)	0.005 (3)
C20	0.039 (4)	0.031 (3)	0.022 (3)	0.007 (3)	0.007 (3)	0.002 (3)
C21	0.028 (3)	0.031 (3)	0.018 (3)	0.002 (3)	0.006 (2)	0.001 (3)
P4	0.0187 (7)	0.0196 (7)	0.0192 (7)	−0.0035 (6)	0.0051 (6)	0.0010 (6)
C22	0.021 (3)	0.020 (3)	0.030 (3)	−0.007 (2)	0.007 (2)	−0.005 (2)
C23	0.027 (3)	0.035 (4)	0.032 (3)	0.001 (3)	0.008 (3)	−0.003 (3)
C24	0.046 (4)	0.022 (3)	0.059 (4)	−0.004 (3)	0.011 (3)	−0.014 (3)
C25	0.028 (3)	0.036 (4)	0.049 (4)	0.002 (3)	0.011 (3)	−0.021 (3)
C26	0.069 (5)	0.040 (4)	0.042 (4)	−0.009 (3)	0.029 (3)	−0.009 (3)
C27	0.071 (5)	0.024 (3)	0.040 (4)	−0.009 (3)	0.032 (3)	−0.006 (3)
C28	0.021 (3)	0.019 (3)	0.019 (3)	−0.006 (2)	0.006 (2)	0.002 (2)
C29	0.026 (3)	0.042 (4)	0.026 (3)	−0.002 (3)	0.007 (2)	0.008 (3)
C30	0.026 (3)	0.049 (4)	0.037 (3)	−0.010 (3)	0.015 (3)	0.003 (3)
C31	0.039 (4)	0.036 (3)	0.022 (3)	−0.008 (3)	0.008 (3)	0.001 (3)
C32	0.034 (4)	0.052 (4)	0.039 (4)	0.016 (3)	0.012 (3)	0.020 (3)
C33	0.021 (3)	0.054 (4)	0.034 (3)	0.012 (3)	0.015 (3)	0.015 (3)
C34	0.015 (3)	0.029 (3)	0.028 (3)	−0.005 (2)	0.011 (2)	0.015 (3)
C35	0.031 (3)	0.048 (4)	0.026 (3)	0.013 (3)	0.003 (3)	0.009 (3)
C36	0.048 (4)	0.056 (4)	0.047 (4)	0.028 (4)	0.014 (4)	0.018 (4)
C37	0.032 (4)	0.085 (6)	0.053 (5)	0.017 (4)	0.009 (3)	0.040 (4)
C38	0.022 (4)	0.088 (6)	0.037 (4)	−0.015 (4)	−0.011 (3)	0.033 (4)
C39	0.028 (3)	0.044 (4)	0.035 (3)	−0.017 (3)	0.000 (3)	0.014 (3)
O100	0.061 (4)	0.110 (5)	0.081 (4)	0.038 (3)	0.031 (3)	0.018 (4)
C101	0.094 (7)	0.081 (6)	0.062 (5)	−0.012 (5)	0.019 (5)	0.002 (5)
C102	0.051 (5)	0.062 (5)	0.068 (5)	−0.002 (4)	0.004 (4)	0.006 (4)
C103	0.057 (5)	0.090 (6)	0.062 (5)	0.015 (4)	0.022 (4)	0.006 (5)
C104	0.049 (5)	0.082 (6)	0.056 (5)	0.005 (4)	0.011 (4)	0.018 (4)

### Geometric parameters (Å, º)

**Table:**

Pd1—C2	2.021 (6)	C20—H20	0.9500
Pd1—C1	2.022 (6)	C21—H21	0.9500
Pd1—P3	2.2998 (15)	P4—C22	1.821 (5)
Pd1—Pd2	2.7381 (8)	P4—C34	1.832 (5)
Pd1—Pd2^i^	2.7446 (9)	P4—C28	1.837 (5)
Pd1—Pd1^i^	3.1704 (14)	C22—C23	1.380 (8)
Pd2—C3	2.095 (6)	C22—C27	1.383 (7)
Pd2—C2	2.154 (5)	C23—C24	1.395 (8)
Pd2—C1^i^	2.169 (6)	C23—H23	0.9500
Pd2—P4	2.3208 (15)	C24—C25	1.365 (9)
Pd2—Pd1^i^	2.7446 (9)	C24—H24	0.9500
Pd2—Pd2^i^	2.8006 (12)	C25—C26	1.358 (8)
C1—O1	1.159 (6)	C25—H25	0.9500
C1—Pd2^i^	2.169 (6)	C26—C27	1.377 (8)
C2—O2	1.164 (6)	C26—H26	0.9500
C3—O3	1.178 (9)	C27—H27	0.9500
C3—Pd2^i^	2.095 (6)	C28—C33	1.373 (7)
P3—C4	1.827 (5)	C28—C29	1.384 (7)
P3—C16	1.830 (5)	C29—C30	1.388 (8)
P3—C10	1.836 (5)	C29—H29	0.9500
C4—C5	1.385 (7)	C30—C31	1.377 (8)
C4—C9	1.392 (7)	C30—H30	0.9500
C5—C6	1.373 (7)	C31—C32	1.369 (8)
C5—H5	0.9500	C31—H31	0.9500
C6—C7	1.387 (7)	C32—C33	1.394 (8)
C6—H6	0.9500	C32—H32	0.9500
C7—C8	1.368 (8)	C33—H33	0.9500
C7—H7	0.9500	C34—C35	1.391 (8)
C8—C9	1.372 (8)	C34—C39	1.391 (7)
C8—H8	0.9500	C35—C36	1.375 (8)
C9—H9	0.9500	C35—H35	0.9500
C10—C11	1.374 (7)	C36—C37	1.377 (10)
C10—C15	1.401 (7)	C36—H36	0.9500
C11—C12	1.390 (7)	C37—C38	1.361 (10)
C11—H11	0.9500	C37—H37	0.9500
C12—C13	1.388 (8)	C38—C39	1.395 (9)
C12—H12	0.9500	C38—H38	0.9500
C13—C14	1.379 (8)	C39—H39	0.9500
C13—H13	0.9500	O100—C101	1.410 (9)
C14—C15	1.382 (7)	O100—C104	1.425 (9)
C14—H14	0.9500	C101—C102	1.512 (11)
C15—H15	0.9500	C101—H10A	0.9900
C16—C21	1.385 (7)	C101—H10B	0.9900
C16—C17	1.394 (7)	C102—C103	1.503 (10)
C17—C18	1.389 (8)	C102—H10C	0.9900
C17—H17	0.9500	C102—H10D	0.9900
C18—C19	1.376 (8)	C103—C104	1.490 (10)
C18—H18	0.9500	C103—H10E	0.9900
C19—C20	1.379 (8)	C103—H10F	0.9900
C19—H19	0.9500	C104—H10G	0.9900
C20—C21	1.384 (7)	C104—H10H	0.9900
			
C2—Pd1—C1	150.4 (2)	C17—C18—H18	119.4
C2—Pd1—P3	98.89 (15)	C18—C19—C20	118.3 (5)
C1—Pd1—P3	102.22 (16)	C18—C19—H19	120.9
C2—Pd1—Pd2	51.16 (15)	C20—C19—H19	120.9
C1—Pd1—Pd2	111.46 (15)	C19—C20—C21	121.4 (5)
P3—Pd1—Pd2	146.24 (4)	C19—C20—H20	119.3
C2—Pd1—Pd2^i^	111.07 (15)	C21—C20—H20	119.3
C1—Pd1—Pd2^i^	51.44 (15)	C20—C21—C16	120.4 (5)
P3—Pd1—Pd2^i^	149.92 (4)	C20—C21—H21	119.8
Pd2—Pd1—Pd2^i^	61.43 (3)	C16—C21—H21	119.8
C2—Pd1—Pd1^i^	92.97 (15)	C22—P4—C34	102.1 (2)
C1—Pd1—Pd1^i^	92.60 (15)	C22—P4—C28	105.2 (2)
P3—Pd1—Pd1^i^	123.13 (4)	C34—P4—C28	103.0 (2)
Pd2—Pd1—Pd1^i^	54.768 (18)	C22—P4—Pd2	112.06 (17)
Pd2^i^—Pd1—Pd1^i^	54.58 (2)	C34—P4—Pd2	116.44 (17)
C3—Pd2—C2	126.58 (14)	C28—P4—Pd2	116.45 (16)
C3—Pd2—C1^i^	126.77 (14)	C23—C22—C27	117.8 (5)
C2—Pd2—C1^i^	102.29 (19)	C23—C22—P4	125.1 (4)
C3—Pd2—P4	99.66 (15)	C27—C22—P4	117.0 (4)
C2—Pd2—P4	94.64 (15)	C22—C23—C24	120.0 (6)
C1^i^—Pd2—P4	95.57 (15)	C22—C23—H23	120.0
C3—Pd2—Pd1	97.67 (12)	C24—C23—H23	120.0
C2—Pd2—Pd1	46.94 (15)	C25—C24—C23	121.4 (6)
C1^i^—Pd2—Pd1	102.24 (15)	C25—C24—H24	119.3
P4—Pd2—Pd1	140.12 (4)	C23—C24—H24	119.3
C3—Pd2—Pd1^i^	97.47 (12)	C26—C25—C24	118.5 (6)
C2—Pd2—Pd1^i^	102.83 (14)	C26—C25—H25	120.8
C1^i^—Pd2—Pd1^i^	46.81 (15)	C24—C25—H25	120.8
P4—Pd2—Pd1^i^	140.88 (4)	C25—C26—C27	121.2 (6)
Pd1—Pd2—Pd1^i^	70.66 (3)	C25—C26—H26	119.4
C3—Pd2—Pd2^i^	48.05 (15)	C27—C26—H26	119.4
C2—Pd2—Pd2^i^	104.98 (15)	C26—C27—C22	121.2 (6)
C1^i^—Pd2—Pd2^i^	104.73 (15)	C26—C27—H27	119.4
P4—Pd2—Pd2^i^	147.71 (4)	C22—C27—H27	119.4
Pd1—Pd2—Pd2^i^	59.40 (2)	C33—C28—C29	118.8 (5)
Pd1^i^—Pd2—Pd2^i^	59.171 (19)	C33—C28—P4	118.3 (4)
O1—C1—Pd1	142.6 (4)	C29—C28—P4	122.8 (4)
O1—C1—Pd2^i^	135.0 (4)	C28—C29—C30	120.4 (5)
Pd1—C1—Pd2^i^	81.7 (2)	C28—C29—H29	119.8
O2—C2—Pd1	140.6 (4)	C30—C29—H29	119.8
O2—C2—Pd2	136.5 (4)	C31—C30—C29	120.2 (5)
Pd1—C2—Pd2	81.9 (2)	C31—C30—H30	119.9
O3—C3—Pd2	138.05 (15)	C29—C30—H30	119.9
O3—C3—Pd2^i^	138.05 (15)	C32—C31—C30	119.6 (5)
Pd2—C3—Pd2^i^	83.9 (3)	C32—C31—H31	120.2
C4—P3—C16	104.9 (2)	C30—C31—H31	120.2
C4—P3—C10	103.7 (2)	C31—C32—C33	120.1 (5)
C16—P3—C10	104.2 (2)	C31—C32—H32	119.9
C4—P3—Pd1	113.63 (16)	C33—C32—H32	119.9
C16—P3—Pd1	113.95 (16)	C28—C33—C32	120.7 (5)
C10—P3—Pd1	115.24 (17)	C28—C33—H33	119.6
C5—C4—C9	118.0 (5)	C32—C33—H33	119.6
C5—C4—P3	123.6 (4)	C35—C34—C39	117.4 (5)
C9—C4—P3	118.4 (4)	C35—C34—P4	120.2 (4)
C6—C5—C4	121.4 (5)	C39—C34—P4	122.4 (4)
C6—C5—H5	119.3	C36—C35—C34	122.1 (6)
C4—C5—H5	119.3	C36—C35—H35	119.0
C5—C6—C7	119.4 (5)	C34—C35—H35	119.0
C5—C6—H6	120.3	C35—C36—C37	119.5 (7)
C7—C6—H6	120.3	C35—C36—H36	120.3
C8—C7—C6	120.0 (5)	C37—C36—H36	120.3
C8—C7—H7	120.0	C38—C37—C36	120.2 (6)
C6—C7—H7	120.0	C38—C37—H37	119.9
C7—C8—C9	120.4 (5)	C36—C37—H37	119.9
C7—C8—H8	119.8	C37—C38—C39	120.6 (6)
C9—C8—H8	119.8	C37—C38—H38	119.7
C8—C9—C4	120.8 (5)	C39—C38—H38	119.7
C8—C9—H9	119.6	C34—C39—C38	120.3 (6)
C4—C9—H9	119.6	C34—C39—H39	119.8
C11—C10—C15	120.1 (5)	C38—C39—H39	119.8
C11—C10—P3	123.7 (4)	C101—O100—C104	109.6 (6)
C15—C10—P3	116.1 (4)	O100—C101—C102	106.6 (7)
C10—C11—C12	120.1 (5)	O100—C101—H10A	110.4
C10—C11—H11	120.0	C102—C101—H10A	110.4
C12—C11—H11	120.0	O100—C101—H10B	110.4
C13—C12—C11	119.7 (5)	C102—C101—H10B	110.4
C13—C12—H12	120.1	H10A—C101—H10B	108.6
C11—C12—H12	120.1	C103—C102—C101	101.7 (6)
C14—C13—C12	120.3 (5)	C103—C102—H10C	111.4
C14—C13—H13	119.8	C101—C102—H10C	111.4
C12—C13—H13	119.8	C103—C102—H10D	111.4
C13—C14—C15	120.1 (6)	C101—C102—H10D	111.4
C13—C14—H14	119.9	H10C—C102—H10D	109.3
C15—C14—H14	119.9	C104—C103—C102	104.0 (6)
C14—C15—C10	119.6 (5)	C104—C103—H10E	111.0
C14—C15—H15	120.2	C102—C103—H10E	111.0
C10—C15—H15	120.2	C104—C103—H10F	111.0
C21—C16—C17	118.4 (5)	C102—C103—H10F	111.0
C21—C16—P3	123.0 (4)	H10E—C103—H10F	109.0
C17—C16—P3	118.5 (4)	O100—C104—C103	107.0 (6)
C18—C17—C16	120.2 (5)	O100—C104—H10G	110.3
C18—C17—H17	119.9	C103—C104—H10G	110.3
C16—C17—H17	119.9	O100—C104—H10H	110.3
C19—C18—C17	121.2 (5)	C103—C104—H10H	110.3
C19—C18—H18	119.4	H10G—C104—H10H	108.6
			
C16—P3—C4—C5	−105.5 (4)	C34—P4—C22—C27	63.2 (5)
C10—P3—C4—C5	3.6 (5)	C28—P4—C22—C27	170.4 (4)
Pd1—P3—C4—C5	129.5 (4)	Pd2—P4—C22—C27	−62.1 (5)
C16—P3—C4—C9	77.1 (4)	C27—C22—C23—C24	0.0 (8)
C10—P3—C4—C9	−173.9 (4)	P4—C22—C23—C24	−179.8 (4)
Pd1—P3—C4—C9	−48.0 (4)	C22—C23—C24—C25	−1.2 (9)
C9—C4—C5—C6	1.9 (8)	C23—C24—C25—C26	1.3 (9)
P3—C4—C5—C6	−175.5 (4)	C24—C25—C26—C27	−0.3 (10)
C4—C5—C6—C7	−1.5 (8)	C25—C26—C27—C22	−0.9 (10)
C5—C6—C7—C8	−0.1 (9)	C23—C22—C27—C26	1.0 (9)
C6—C7—C8—C9	1.2 (9)	P4—C22—C27—C26	−179.2 (5)
C7—C8—C9—C4	−0.7 (9)	C22—P4—C28—C33	75.3 (5)
C5—C4—C9—C8	−0.8 (8)	C34—P4—C28—C33	−178.1 (4)
P3—C4—C9—C8	176.7 (4)	Pd2—P4—C28—C33	−49.4 (5)
C4—P3—C10—C11	−99.7 (5)	C22—P4—C28—C29	−106.7 (5)
C16—P3—C10—C11	9.8 (5)	C34—P4—C28—C29	−0.1 (5)
Pd1—P3—C10—C11	135.4 (4)	Pd2—P4—C28—C29	128.6 (4)
C4—P3—C10—C15	77.9 (4)	C33—C28—C29—C30	−0.5 (8)
C16—P3—C10—C15	−172.5 (4)	P4—C28—C29—C30	−178.6 (4)
Pd1—P3—C10—C15	−46.9 (4)	C28—C29—C30—C31	−2.3 (9)
C15—C10—C11—C12	0.0 (8)	C29—C30—C31—C32	2.7 (9)
P3—C10—C11—C12	177.5 (4)	C30—C31—C32—C33	−0.4 (9)
C10—C11—C12—C13	−2.3 (8)	C29—C28—C33—C32	2.8 (9)
C11—C12—C13—C14	2.5 (8)	P4—C28—C33—C32	−179.1 (5)
C12—C13—C14—C15	−0.3 (8)	C31—C32—C33—C28	−2.4 (10)
C13—C14—C15—C10	−2.1 (8)	C22—P4—C34—C35	−156.5 (4)
C11—C10—C15—C14	2.2 (8)	C28—P4—C34—C35	94.6 (4)
P3—C10—C15—C14	−175.5 (4)	Pd2—P4—C34—C35	−34.1 (5)
C4—P3—C16—C21	−4.3 (5)	C22—P4—C34—C39	24.4 (5)
C10—P3—C16—C21	−113.0 (4)	C28—P4—C34—C39	−84.5 (5)
Pd1—P3—C16—C21	120.6 (4)	Pd2—P4—C34—C39	146.8 (4)
C4—P3—C16—C17	178.4 (4)	C39—C34—C35—C36	0.7 (8)
C10—P3—C16—C17	69.7 (4)	P4—C34—C35—C36	−178.5 (5)
Pd1—P3—C16—C17	−56.7 (4)	C34—C35—C36—C37	−1.0 (9)
C21—C16—C17—C18	0.9 (7)	C35—C36—C37—C38	0.5 (10)
P3—C16—C17—C18	178.3 (4)	C36—C37—C38—C39	0.3 (10)
C16—C17—C18—C19	−0.9 (7)	C35—C34—C39—C38	0.2 (8)
C17—C18—C19—C20	0.9 (7)	P4—C34—C39—C38	179.3 (4)
C18—C19—C20—C21	−0.8 (7)	C37—C38—C39—C34	−0.6 (9)
C19—C20—C21—C16	0.8 (7)	C104—O100—C101—C102	16.7 (9)
C17—C16—C21—C20	−0.8 (7)	O100—C101—C102—C103	−30.5 (8)
P3—C16—C21—C20	−178.1 (4)	C101—C102—C103—C104	32.2 (8)
C34—P4—C22—C23	−117.1 (5)	C101—O100—C104—C103	4.4 (9)
C28—P4—C22—C23	−9.8 (5)	C102—C103—C104—O100	−23.6 (8)
Pd2—P4—C22—C23	117.6 (4)		

Symmetry code: (i) −*x*, *y*, −*z*+1/2.

### Hydrogen-bond geometry (Å, º)

**Table:**

*D*—H···*A*	*D*—H	H···*A*	*D*···*A*	*D*—H···*A*
C19—H19···O100^ii^	0.95	2.43	3.282 (8)	149

Symmetry code: (ii) *x*−1/2, *y*+1/2, *z*.
